# Post COVID-19 vaccination coverage recovery model

**DOI:** 10.1186/s13052-024-01777-9

**Published:** 2024-11-25

**Authors:** Marina Giuliana Del Piano, Marina Russo, Francesca Vassallo, Pietro Buono, Tiziana Ciarambino, Carmine Carbone, Giuseppe Russo

**Affiliations:** 1 Complex Unit of Epidemiology and Prevention Local Health Agency Na3 Sud, University of Naples Federico II, Brusciano, 167 80053 Castellammare di Stabia (Na), Piazza S. Giovanni, 80031 Italy; 2 Department of Translational Medical Sciences, Section of Pediatrics, University of Naples Federico II, Via Sergio Pansini, 5, Napoli, 80131 Italy; 3 UOD Consultancy Activies and Maternal and Child Care- Management Center Is. C3 80143 Napoli General Direction for Health Protection and Coordination of the Regional Health System, Campania, Italy; 4 Prevention Department Local Health Agency Na3 Sud- C.so A. de Gasperi, Castellammare di Stabia, 167 80053 Italy; 5 General Director Local Health Agency Na3 Sud , via Marconi, Torre del Greco, 66-80059 Italy

**Keywords:** Vaccination coverage, Public health policy, Tetanus-diphtheria- pertussis, Hepatits B, Haemophilus influenzae type b, Measles, Pneumococcal disease, Rotavirus, Chickenpox

## Abstract

Vaccination is a crucial tool for the primary prevention of infectious diseases. Thanks to the widespread of vaccinations it has been possible to eradicate very serious diseases. Unfortunately, vaccination coverage in Italy has been decreasing starting from 2015, getting worse during COVID-19. Nowadays, very few Italian regions have achieved the goal of 95% coverage. In this study we present a vaccination recovery model proposed by Local Health Department “Napoli 3 Sud” in Campania. An evaluation of the vaccination coverage from January 2019 to December 2021 of the 13 Districts of the Local Health Department “Napoli 3 Sud” in Campania was carried out, by extraction from the regional computerized platform “GE.VA” Regional Vaccine Registry and from Sinfonia Vaccini Soresa platform. Vaccination coverage of the Local Health Department “Napoli 3 Sud” for the cohorts of newborns in the year 2019–2021 improved to an average of 96.29% for Pneumococco, of 84.78% for Meningococcal, of 94.3% for Measles, Mumps and Rubella, 91.4% for chickenpox. This study highlights how the collaboration between family pediatricians and the Local Health Department, with the help of a regional computerized platform GE.VA, is effective in improving vaccination coverage.

## Introduction

Vaccinations represent one of the key elements of preventive medicine, as they have substantially modified the natural history of numerous infectious diseases. Thanks to mass immunization policies, it has been possible to eradicate very serious diseases, such as Tetanus and Polio, which in the past have been responsible for millions of deaths and permanent disabilities [1].

Vaccinations are therefore considered a true “collective intervention”, because they reduce individual susceptibility to infection, through the control of transmission [2]. The extraordinary nature of the instrument is given by the fact that, despite a modest use of resources, it brings large benefits in terms of both individual and collective immunity [3].

The World Health Organization recommends that the immunization threshold of the population must be no less than 95%, in order to achieve the so-called “herd immunity” [4]. However, starting from 2015, there was a progressive and dangerous decline in national vaccination coverage, especially for some pathologies such as Measles and Rubella for which between 2013 and 2015 even 5% points were lost [5]. Indeed, in 2017 there was a significant outbreak of measles in Italy, due to a large pool of measles-susceptible people. The reduction in coverage was further exacerbated by the SARS-CoV2 pandemic, due to the fear of contagion and the closure of some vaccination services [6]. This low vaccination coverage, on one hand, created pockets of susceptible subjects and, on the other hand, prolonged the time to recover the community vaccination coverage. Based on this recent data, a recovery plan was mandatory. Thus, 13 districts of Local Health Department “Napoli 3 Sud” in Campania started an active collaboration with family pediatricians of the same Local Health Department focused on vaccination process management with aim of increasing vaccination coverage.

## Methods

An evaluation of the vaccination coverage of the 13 Local Health Department “Napoli 3 Sud” in Campania was carried out from 2019 to 2021, including children borned from 2018 to 2020. The 13 districts were Number 34 Portici, Number 48 Marigliano, Number 49 Nola, Number 50 Volla, Number 51 Pomigliano d’Arco, Number 52 Palma Campania,, Number 53 Castellammare, Number 54 San Giorgio a Cremano and Number 55 Ercolano, Number 56 Torre Annunziata, Number 57 Torre del Greco, Number 58 Pompei. We compared the average of this 13 districts vaccination coverage with the percentage of coverage of all Campania region. Data were collected from regional computerized platform GEVA (Regional Vaccine Registry) and Sinfonia Vaccini Soresa platform. So.Re.Sa S.p.A. provides this platform that is an informatic support of the Campania Region for planning health, economic and management activities as well as providing analytical evidence to make the monitoring and evaluation of the Regional Health Service Performance. The IT platform for registering vaccines, of the Vaccinal Registry in Campania, allowed a verification of the vaccines actually administered. A statistical software (SPSS) permitted the extraction of data based on specific age cohorts, by individual district and local health authority.

Data of non compliant children was collected and family paediatricians were informed. Patients were called back for visits at their offices. During the visit the importance of vaccines was explained and they were encouraged to reschedule their appointment to vaccination centerData relating to vaccination activities and coverage achieved for polio, diphtheria, tetanus, pertussis, hepatitis B, invasive infections from Haemophilus influenzae type b (Hib) (Hexavalent), Pneumococcus (PCV 13), measles, mumps and rubella are annually sent to the Ministry of Health using a specially prepared detection card. The data covered by this work derive from extractions provided by the Sinfonia Vaccini digital platform in Campania.

## Results

The report of vaccination coverage for the cohorts of newborns in the year 2018 (at the age of 36 months in 2021) and for newborns in the year 2020 (at the age of 36 months in 2023), of the Local Health Department “Napoli 3 Sud” with its 13 districts, showed an optimal coverage trend. This data were collected at the end of 2019, 2020 and 2021, respectively. Thanks to this coverage recovery model, based on the recall of non vaccinated children, in the first year of life, coverage with 3 complete doses of hexavalent vaccine from an average of 80% in the 2018 birth cohort to an average of 83.7% in the subsequent 2020 birth cohort. Rotavirus coverage went from an average of 47.7% of the 2018 birth cohort to an average of 73.71% of the 2020 birth cohort. Again, in the first year of life, coverage for Pneumococcus improved from an average of 76.1% of the 2018 birth cohort to an average of 96.29% of the 2020 birth cohort (Table 1). More in details, results for improvement in the 13 districts for Hexavalent, MPR, Pneumococcus and Meningococcal B and ACWY (MEN B, MEN ACWY) are showed in Tables 2, 3, 4 and 5.

**Table 1:**
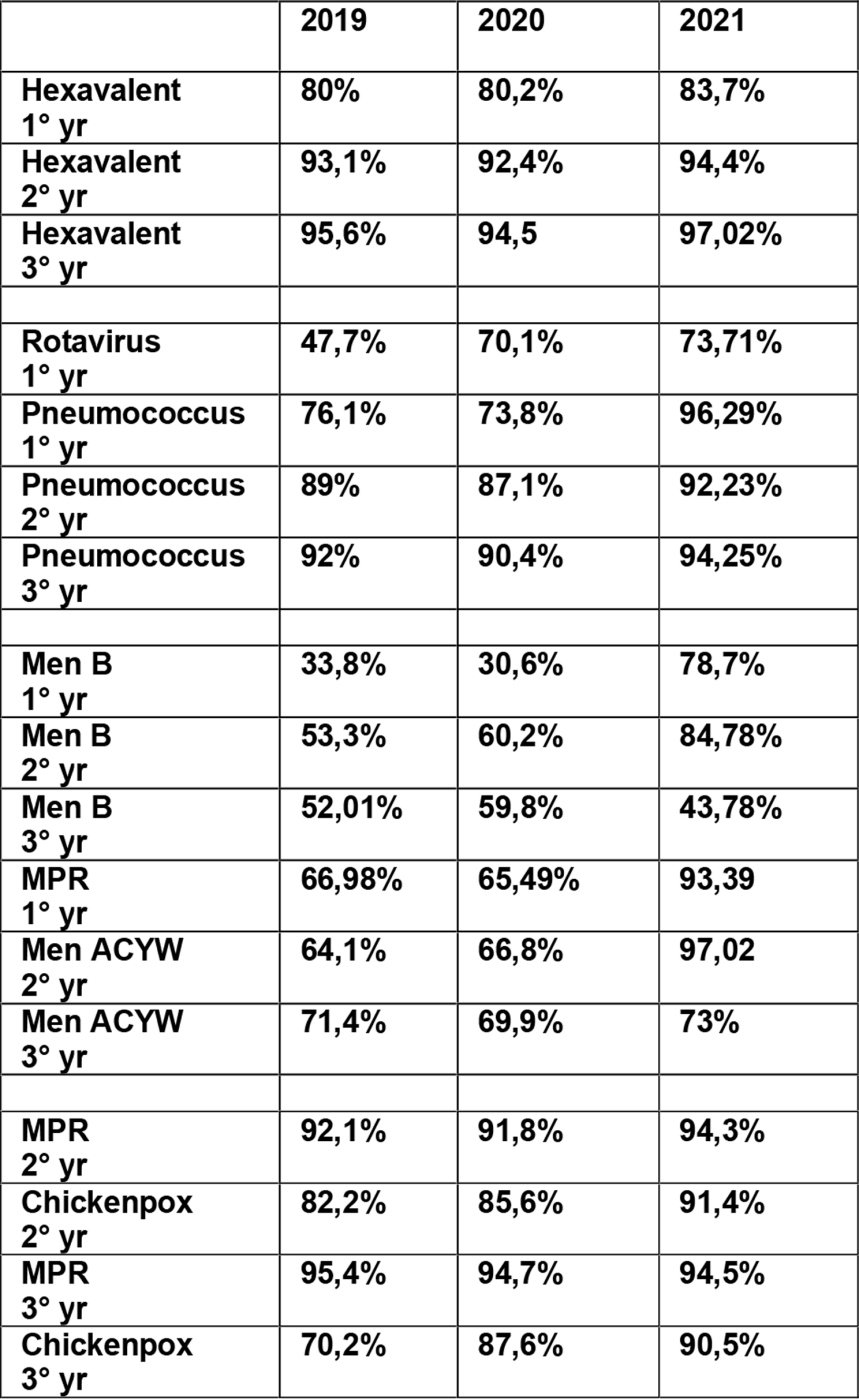
Results of vaccination coverage for the cohorts 2018–2020,data detected at the end of 2019,2020 and 2021 respectively, Local Health Department “Napoli 3 Sud”

**Table 2:** Vaccination coverage for hexavalent in the first year of life

Districts	2019	2020	2021
	%	%	%
DS 34	84.9%	83.8	86.6
DS 48	82.8	82.2	82.7
DS 49	86.1	86.0	87.4
DS 50	85.9	81.4	85.3
DS 51	86.9	87.7	91.3
DS 52	79.6	87,1%	91.3
DS 53	74.2	76.2	83.2
DS 54	77.4	75.1	86.2
DS 55	66.7	70.9	68.2
DS 56	60.1	61.7	66.1
DS 57	76.4	77.2	84.9
DS 58	82.8	82.0	87.5
DS 59	91.5	91.1	91.6
ALS	80.0	80.2	83.7

**Table 3:** Vaccination coverage for rotavirus in the first year of life

Districts	2019	2020	2021
	%	%	%
DS 34	41.3	61.7	74.3
DS 48	41.0	59.6	73.3
DS 49	42.2	80.2	86.9
DS 50	61.5	73.7	66.4
DS 51	69.5	86.0	88.0
DS 52	58.8	76.6	84.2
DS 53	23.0	63.2	78.5
DS 54	71.5	83.8	86.5
DS 55	69.7	75.3	73.3
DS 56	23.0	46.7	52.8
DS 57	45.6	72.6	82.8
DS 58	32.9	58.9	63.3
DS 59	60.7	84.4	84.1
ASL	47.7	70.1	76.1

**Table 4:** Vaccination coverage for pneumoccocus in the first year of life

Districts	2019	2020	2021
	%	%	%
DS 34	81.9	77.6	81.3
DS 48	72.2	73.1	78.7
DS 49	85.6	83.8	84.7
DS 50	84.7	80.0	82.9
DS 51	80.9	84.6	88.3
DS 52	78.9	79.6	82.4
DS 53	72.6	75.2	81.6
DS 54	76.2	70.0	82.4
DS 55	63.8	65.6	63.1
DS 56	57.1	51.7	54.7
DS 57	62.8	57.4	67.5
DS 58	80.2	71.9	82.9
DS 59	89.2	87.6	89.2
ASL	76.1	73.8	78.7

**Table 5:** Vaccination coverage for menigococcus B in the first year of life

Districts	2019	2020	2021
	%	%	%
DS 34	41.6	45.0	46.4
DS 48	35.1	31.1	39.1
DS 49	40.6	40.7	28.6
DS 50	43.6	16.7	18.8
DS 51	52.8	42.9	38.7
DS 52	22.5	26.7	24.0
DS 53	30.0	24.8	30.1
DS 54	22.3	16.7	21.0
DS 55	17.2	23.3	24.3
DS 56	21.6	21.1	20.8
DS 57	34.3	37.3	33.8
DS 58	40.6	31.4	26.3
DS 59	36.8	38.7	36.9
ASL	33.8	30.6	29.4

In the second year of life, hexavalent vaccination coverage went from an average of 93.1% of the 2018 birth cohort to an average of 94.4% of the 2021 birth cohort; that for Meningococcal B (complete cycle) went from an average of 53.3% in 2018 to an average of 84.78% in 2020. Furthermore, an improvement was also recorded in the average coverage for Meningococcal ACWY, which went from 64.1% of the 2018 birth cohort to 97.02% of the 2020 birth cohort, and in that for Measles, Mumps and Rubella (MMR) which went from an average of 92.1% of the 2018 birth cohort to 94.3% of the 2020 birth cohort, while for Chickenpox it increased from an average value of 82.2% of the 2018 birth cohort to 91.4% of the 2020 birth cohort (Table 1).

At the age of three, there was an increase in the average coverage for chickenpox (Table 1).

**Fig. 1:**
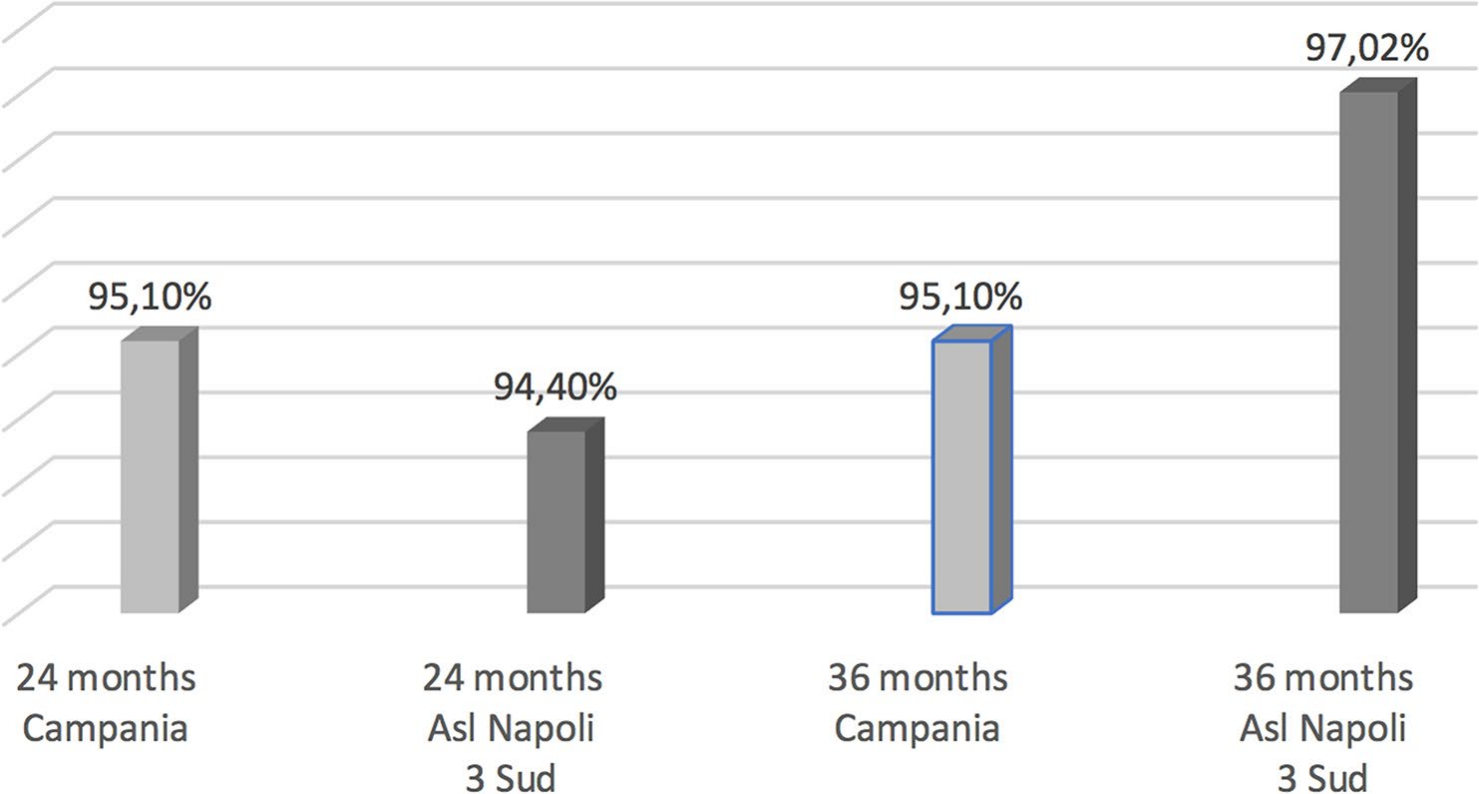
Hexavalent vaccination coverage cohort 2018

**Fig. 2:**
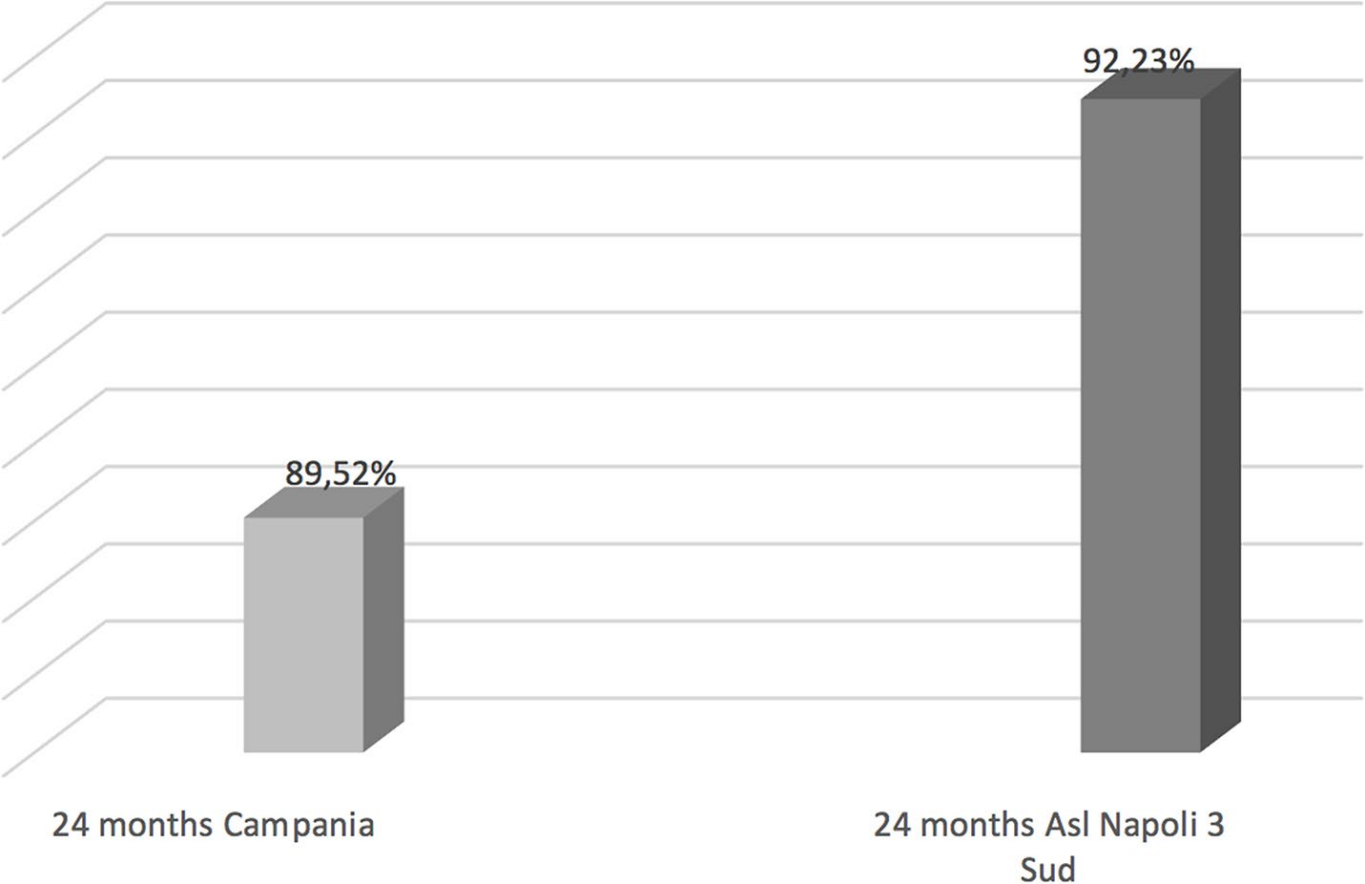
Pneumococcus vaccination coverage cohort 2018

**Fig. 3:**
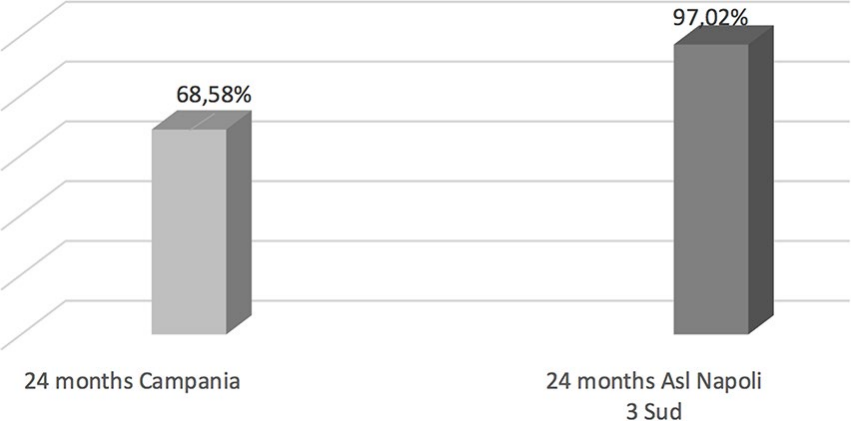
MEN C vaccination coverage cohort 2018

**Fig. 4:**
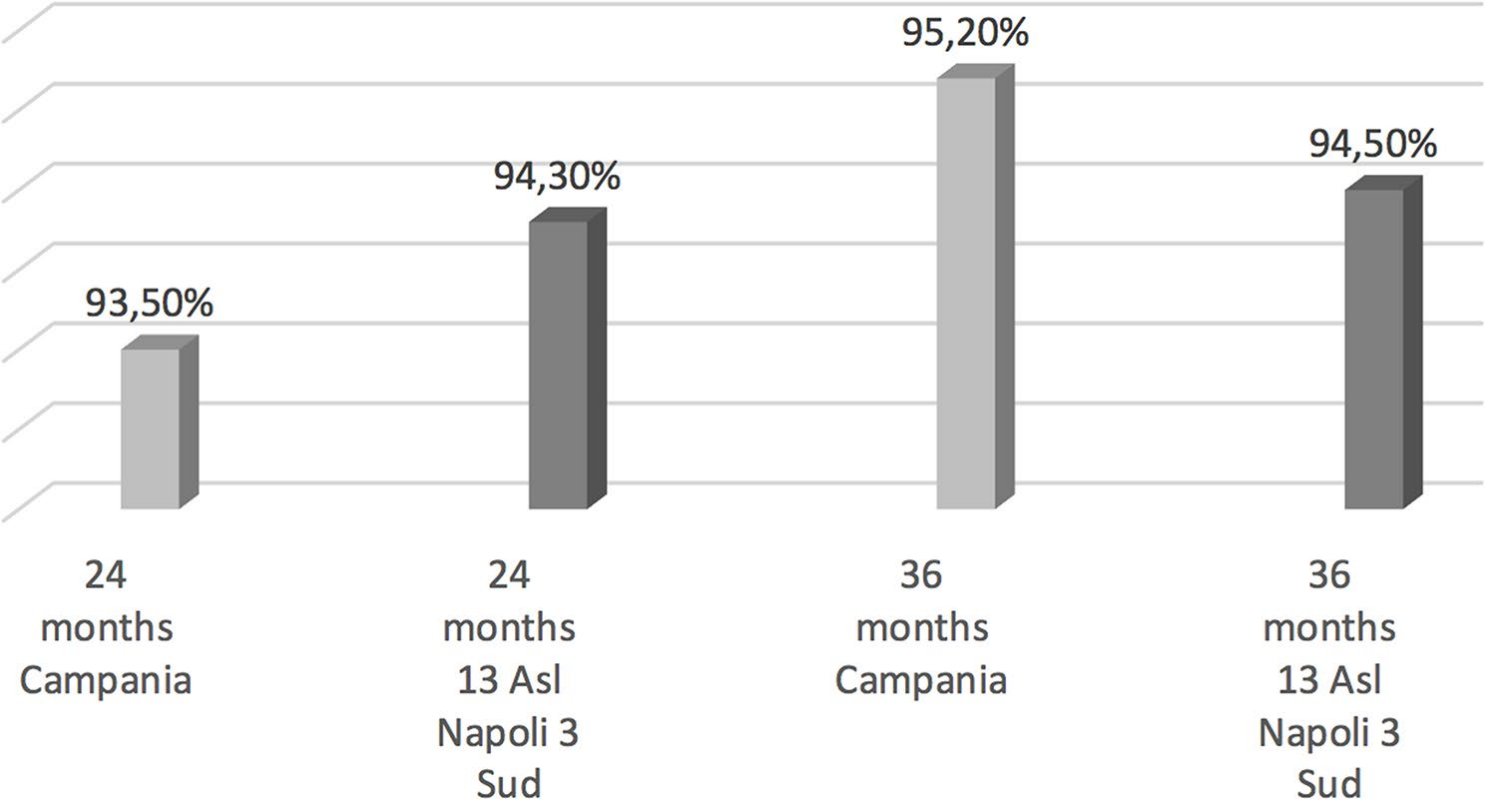
MPR vaccination coverage cohort 2018

Compared to the coverage of all Campania region we observed for hexavalent an increase of rates at 36 months (Fig. 1), for pneumococcus at 24 months of life (Fig. 2) and for Meningococcus C at 24 months (Fig. 3). While data concerning MMR and Measles vaccine are worse comparing to all Campania region (Fig. 4).

## Discussion

The direct observation obtained from the analysis of the vaccinated cohorts allows us to verify the effectiveness of the recovery vaccination program proposed by Local Health Department “Napoli 3 Sud”. Registration on the platform automatically provides an answer and guides the decision-making processes.

Until 2016, data on vaccination coverage were published every year at 24 months, representing the proportion of children born in a given year who were adequately vaccinated at the time of the survey (for example, the coverage of children born in 2013 is calculated with the vaccinations completed on 31 December 2015 and calculated in 2016). From 2016 to today, however, the 36-month vaccination coverage is also published to update, after one year, the coverage data relating to the same cohort. This data is useful for evaluating the share of children who, at the previous year’s vaccination survey, were non-compliant and who were recovered. Based on the results published at 12, 24 and 36 months of life, in the three-year period 2019–2021, we can confirm that there has been a significant and progressive improvement in all vaccination coverage indices [7]. In particular, for the hexavalent vaccine against diphtheria, tetanus, pertussis, polio, Haemophylus influenzae type b and hepatitis B and for the vaccine against measles, rubella and mumps, both the 24-month and 36-month data show a vaccination coverage that it stands at an average > 94%, which is in line with the objective of the National Vaccination Plan 2017–2019 and of the new National Vaccination Plan 2023–2025. Furthermore, there was an increase in the coverage rates for Rotavirus at 1 year of life in the 2021 cohort (73.71%) and an increase of 9.2% and 20% in the coverage rates for Chickenpox in the 2nd year and 20% in the 3rd year of life. Based on this data, the herd immunization was reached by Hexavalent, MPR, MEN ACWY and Pneumococcus. The worst coverage in our group was reported for Rotavirus according with F. Napolitano et al. [8], who showed that only 15.3% of parents in Italy declared that they had immunized their children against rotavirus infection. Data regarding each single district reported for the children in their first year of life showed an improvement of coverage for Hexavalent, Rotavirus and Pneumococcus. Among the districts, those with the lowest vaccination coverage were the most rural (Number 53 and Number 56).

As we already know, vaccination is one of the most effective ways to prevent presently existing infectious diseases. It prevents 2–3 million deaths a year; a further 1.5 million could be prevented if global vaccination given; it is one of the most important tools of primary prevention, this should be a voluntary and informed choice. However, with progressive decline in vaccination coverage and particularly the increase in number of measles cases, some countries, such as Italy and France, have decided to enact laws that make vaccinations ‘mandatory’, although with different approaches [9, 10]. In the last century, vaccination was widely accepted across European countries because of the social value of immunization for the protection of individual and collective health [11]. The main task of health care workers (HCWs) is to inform the population about the importance of this preventive measure for the individual and the entire community to obtain social acceptance and, consequently, high voluntary vaccination coverage. The strategic vaccine advisory group of WHO (SAGE) identifies complacency, convenience of accessing vaccines, and lack of confidence as underlying reasons for hesitancy [12]. In our study, the analysis of the vaccinated cohorts show the efficacy of the descripted recovery vaccination model. Vaccination coverage in Italy has been decreasing starting from 2015 getting worse during COVID-19 [13] The decision to enact mandatory laws in some countries (such as Italy, France, and, to a lesser degree, Germany) has already resulted in increase in coverage in the pediatric age. Actually, with the enforcement of national laws, vaccination coverage has increased in both Italy and France. However, making vaccinations mandatory should not be considered a definitive approach but rather a temporary decision to tackle hesitancy, based on the epidemiological situation in each country in order to maintain long-lasting herd immunity effects after reaching optimal levels of immunization coverage. The aim is always to guarantee a protection to the general population, and to avoid the spread of infectious diseases. However, this temporary solution may not suit all contexts, and each country should find the most suitable way to keep up with vaccination coverages according to own cultural and organizational background so is necessary to evaluate new strategies to improve vaccination adherence. Our study confirmed the importance of collaboration between Public Health System and Italian Primary care pediatricians. These results appear very encouraging, also because they show the excellent work carried out during the last three years by these 13 districts of Local Health Department “Napoli 3 Sud” in Campania also based on collaboration with family pediatricians and schools. Although it must be taken into account that it is impossible to extract the complete data on vaccination coverage of the cohort of those born in 2021 at the age of 36 months (3 years of life), as not all those born in 2021 have reached 36 months of life at 31/12/2023, so further date updates are needed. This paper is not without limitations: first of all, we don’t have a reliable percentages of coverage rates prior to the recovery program. Moreover, it will be interesting reporting missing data such as 6 years old children and adolescent, so further studies are needed.

## Data Availability

Not applicable.
